# Smoking and Subclinical ILD in RA versus the Multi-Ethnic Study of Atherosclerosis

**DOI:** 10.1371/journal.pone.0153024

**Published:** 2016-04-06

**Authors:** Cheilonda Johnson, Jon T. Giles, Joan Bathon, David Lederer, Eric A. Hoffman, R. Graham Barr, Sonye K. Danoff

**Affiliations:** 1 Department of Medicine, Division of Pulmonary and Critical Care Medicine, Johns Hopkins University School of Medicine, Baltimore, Maryland, United States of America; 2 Department of Medicine, Division of Rheumatology, Columbia University, College of Physicians & Surgeons, New York, New York, United States of America; 3 Department of Medicine, Division of Pulmonary, Allergy, and Critical Care Medicine, Columbia University, College of Physicians & Surgeons, New York, New York, United States of America; 4 Department of Radiology, University of Iowa Carver College of Medicine, Iowa City, Iowa, United States of America; 5 Department of Medicine, Division of General Medicine, Columbia University, College of Physicians & Surgeons, New York, New York, United States of America; 6 Department of Epidemiology, Columbia University, College of Physicians & Surgeons, New York, New York, United States of America; Medical University of South Carolina, UNITED STATES

## Abstract

A population-based cohort showed an association between cigarette smoking and subclinical parenchymal lung disease defined as regions of increased computed tomography (CT) lung densitometry. This technique has not been applied to the rheumatoid arthritis (RA) population where associated ILD is highly prevalent. The association between cumulative cigarette smoking and volume of areas of high attenuation (HAA: >-600 and <-250 Hounsfield Units) on full inspiratory CT was compared in 172 RA participants and 3,969 controls in a general population sample. Multivariable regression models were used to adjust for demography, anthropometrics, percent emphysema, and CT parameters. The mean cumulative cigarette smoking exposure was 25 (IQR 10–42) and 15(IQR 5–31) pack-years for the RA and non-RA cohorts, respectively. Mean HAA was 153(±57) cm^3^ and 129(±50) cm^3^ in the RA and non-RA cohorts, respectively. Each 10 cigarette pack-year increment was associated with a higher HAA by 0.03% (95% CI, 0.007–0.05%) in RA patients and by 0.008% (95% CI, 0.003–0.01%) in those without RA (interaction *p* = 0.001). Cigarette smoking was associated with higher lung attenuation; with a magnitude of association more pronounced in those with RA than in the general population. These data suggest that cigarette smoking may be a more potent ILD risk factor for RA patients than in the general population.

## Introduction

Interstitial lung disease (ILD) is an important extrarticular manifestation of rheumatoid arthritis(RA)[1]. Since its first description over six decades ago, RA-associated ILD (RA-ILD) remains a significant source of morbidity and mortality [1–4]. This is due in part to poorly understood disease pathobiology and limited therapeutic options[1]. Identifying risk factors is the first step to understanding the mechanisms of this disease and eventually developing more efficacious treatment.

Smoking is a recognized risk factor for the development of RA, particularly among current and former heavy smokers [1, 5–8]. This has led many to hypothesize that smoking is a possible trigger of autoimmunity in RA [9]. Studies evaluating the role of smoking in the development of RA-ILD have shown conflicting results [1, 10, 11]. Some have shown a statistically significant increase in abnormalities on pulmonary function tests (PFT) and radiographic tests [12–14] in smokers with RA, while others have not [10]. While there is evidence that ILD may be statistically more prevalent in smokers with RA, there is no clear temporal or causal relationship between smoking and RA-ILD [15]. Nonetheless, many experts believe that smoking may act synergistically with other host and environmental factors to facilitate the development of ILD among those with RA [15–17]. For example, HLA-DRB1 shared epitope (SE) alleles, a significant genetic risk factor for the development of RA and RA-ILD, show strong gene-environment interactions with smoking [16, 18–20].

High resolution computed tomography (HRCT) is very sensitive at detecting lung abnormalities. In fact, many patients with RA exhibit HRCT evidence of ILD shortly after diagnosis before overt symptoms or PFT abnormalities develop [21, 22]. A large general population based study has shown that cumulative cigarette smoking is a risk factor for high attenuation lung lesions using CT densitometry that could represent preclinical interstitial lung disease [23]. This technique is highly reproducible and has been validated for quantifying the extent of lung parenchyma affected by lung injury, inflammation or fibrosis [23, 24]. Areas of high attenuation empirically correlate with parenchymal inflammation and fibrosis on imaging and restrictive physiology on PFTs [23, 24]. We sought to test the hypothesis that the association of smoking with early evidence of interstitial lung disease based on areas of high lung attenuation on CT would be greater in patients with RA compared with controls without RA.

## Materials and Methods

### Study Populations

Participants were enrolled in two separate but similar cohorts; RA patients in the Evaluation of Subclinical Cardiovascular Disease and Predictors of Events in Rheumatoid Arthritis (ESCAPE RA) Study [25] and non-RA controls in the Multi-Ethnic Study of Atherosclerosis (MESA) [26]. Both prospectively investigated subclinical and progressive subclinical cardiovascular disease and have been previously described [25, 26]. MESA enrolled participants age 45–84 years old between July 2000 and December 2011. Participants were excluded if they had prevalent cardiovascular disease, weighed greater than 300 pounds, or underwent CT examination of the chest within one year of study enrollment. The ESCAPE RA study had the same inclusion/exclusion criteria as MESA, except subjects had to meet American College of Rheumatology RA criteria[27] for at least six months. ESCAPE RA enrolled participants from October 2004 to May 2006. All participants provided institutional review board (IRB) approved written informed consent. MESA was approved by the IRBs of all participating sites (Columbia University, Johns Hopkins University, Northwestern University, UCLA, University of Minnesota, and Wake Forest University) and the National Heart, Lung, and Blood Institute of the National Institutes of Health; the present study was approved by the Johns Hopkins University IRB (NA_32457).

### CT Densitometry

Both groups underwent cardiac multi-detector row computed tomography (MDCT) scanning following the same protocol as part of the Baseline Visit [25, 26]. MESA cardiac MDCT scans of 2.5 to 3.0 mm thickness capture approximately 70% of the lung from the carina to lung bases [28]. CT densitometry[29] is an automated method of CT interpretation where the Hounsfield units (HU) of each individual voxel are determined and summed yielding measures of low and high attenuation areas. Areas of high attenuation (HAA) were defined as voxels between -600 and -250 HU[23]; percent emphysema was defined as the percentage of lung voxels with attenuation less than -910 HU[28, 30]. Both measures are highly correlated with full-lung CT scans in MESA (Spearman correlation coefficient 0.87 for HAA) [23, 28].

### Other Measures

Race/ethnicity were assessed by self-report. Anthropometrics and smoking history were assessed using standardized questionnaires for both groups[26]. Participants were considered ever smokers if they smoked at least 100 cigarettes in their lifetime and current smokers if they smoked in the last 30 days. ESCAPE RA participants had a baseline clinical evaluation including both RA specific and general health questionnaires and were assessed for the presence of the rheumatoid arthritis susceptibility alleles of *HLA-DRB1* (i.e. shared epitope) as previously described [25]. Additionally, ESCAPE RA participants underwent PFTs based on ATS guidelines [31, 32] at a Visit 2 (completed eighteen months after the Baseline Visit).

### Analysis

The volume of high attenuation areas (HAA) was used in the regression models to allow for ease of comparison with published reports and reduce potential spurious correlations [23]. HAA had a natural log-normal distribution which was linear with all continuous variables. For the natural log HAA analysis, multivariable regression models were constructed with potential confounders included that were associated with HAA in univariate analyses at the p<0.20 significance level, to allow for residual confounding. Akaike’s information criterion is used to exclude non-contributory covariates in nested models. The natural log HAA (dependent variable) was regressed on cigarette pack-years (independent variable) after controlling for potential confounders. All calculations were performed using intercooled Stata 12 (StataCorp, College Station, TX). A two-tailed *P* value of less than 0.05 was used as the cutoff for statistical significance; estimates of uncertainty were presented as 95 percent confidence intervals (95% CI).

## Results

CT densitometry data were available for 172 RA and 3,969 and non-RA control participants, respectively. Group participant characteristics are summarized in Table 1. The RA cohort was younger and comprised of primarily Caucasian women. The two groups were anthropometrically similar. Participants with RA were significantly more likely to have smoked and among former smokers, smoked more cigarettes on average. Participants from the RA cohort also had higher values of percent emphysema. Pulmonary characteristics of RA participants at Baseline and Visit 2 are summarized in Table 2. The mean RA duration was eight years. Thirty-eight percent were on prednisone therapy, 87% were on non-biologic DMARDs, and 46% were on biologic DMARDs at the start of the study (Table 2). Forty-one percent had respiratory symptoms with 8% showing a restrictive pattern on PFTs and 17% a diffusing capacity impairment (Table 2). The mean forced vital capacity percent predicted (FVC %) and carbon monoxide diffusing capacity percent predicted (DLCo %) was 101% (Table 2).

**Table 1:** Participant Characteristics.

	MESA Controls (n = 3,969)	ESCAPE RA (n = 172)	*P* Value
**Demographics**			
**Age, years**	61 ± 10	60 ± 9	0.006
**Men, %**	49	41	0.04
**Race/ethnicity**			
White, %	35	88	<0.001
African American, %	26	7	
Asian, %	16	3	
Hispanic, %	23	2	
**Anthropometrics**			
Height, cm	166 ± 10	168 ± 10	0.18
Weight, kg	79 ± 17	80 ± 18	0.21
BMI, kg/m^2^	28.1 ± 5.3	28.4 ± 5.3	0.56
BMI category, %			
<18.5	1	1	0.80
18.5–24.9	29	28	
25–29.9	39	37	
≥30	30	34	
Waist circumference, cm	98 ± 14	96 ± 16	0.14
Hip circumference, cm	105 ± 11	104 ± 14	0.14
**Smoking**			
Never smoker, %	52	38	<0.001
Former smoker, %	36	51	
Current smoker, %	12	11	
**Cigarette pack-years (among ever smokers)**	15 (5–31)	25 (10–42)	<0.001
Percent Emphysema, %*	20 ± 13	34 ± 14	<0.001
**HAA, cm**^**3**^	129 ±50	153 ±57	<0.001

Data are mean ± SD, median (interquartile range), and percentage.

*Percent emphysema is the percentage of total voxels in the whole lung that fell below -910 Hounsfield units.

HAA: High attenuation areas are the volume of total voxels in the whole lung between -600 and -250 Hounsfield units.

**Table 2:** Baseline and Visit 2 RA Patient Characteristics.

**Baseline Characteristics**	**N = 176**
**RA Disease Activity**	
RA Duration, years	8 (4–17)
DAS28-CRP	3.7 (2.9–4.4)
CRP, mg/L	2.4 (1.1–7.7)
IL-6, pg/mL	3.7 (1.8–7.8)
Total SHS	8 (1–37)
**RA Treatment**	
Current prednisone, n (%)	67 (38)
Current non-biologic DMARDs, n (%)	150 (86)
Methotrexate, n (%)	114 (65)
Leflunomide, n (%)	19 (11)
Current biologic DMARDs, n (%)	81 (46)
TNF inhibitor, n (%)	78 (45)
**Visit 2 Characteristics**	**N = 142**
**Pulmonary Function Tests**	
Any abnormality, n (%)	38 (27)
Obstructive PFT pattern, n (%)	14 (10)
Restrictive PFT pattern, n (%)	12 (8)
Impaired Diffusion pattern, n (%)	24 (17)
Isolated impaired diffusion, n (%)	12 (9)
FVC (% predicted)	101 ± 19
DLCO (% predicted)	101 ± 59
**Respiratory Symptoms**	
Any respiratory symptoms, n (%)	69 (41)
Any cough, n (%)	37 (22)
Any breathlessness, n (%)	36 (21)

Data are mean ± SD, median (interquartile range) unless otherwise indicated.

RA = rheumatoid arthritis; DAS = disease activity score; CRP = C-reactive protein; IL = interleukin; SHS = Sharp-van-Heijde Score.

**Fig 1:**
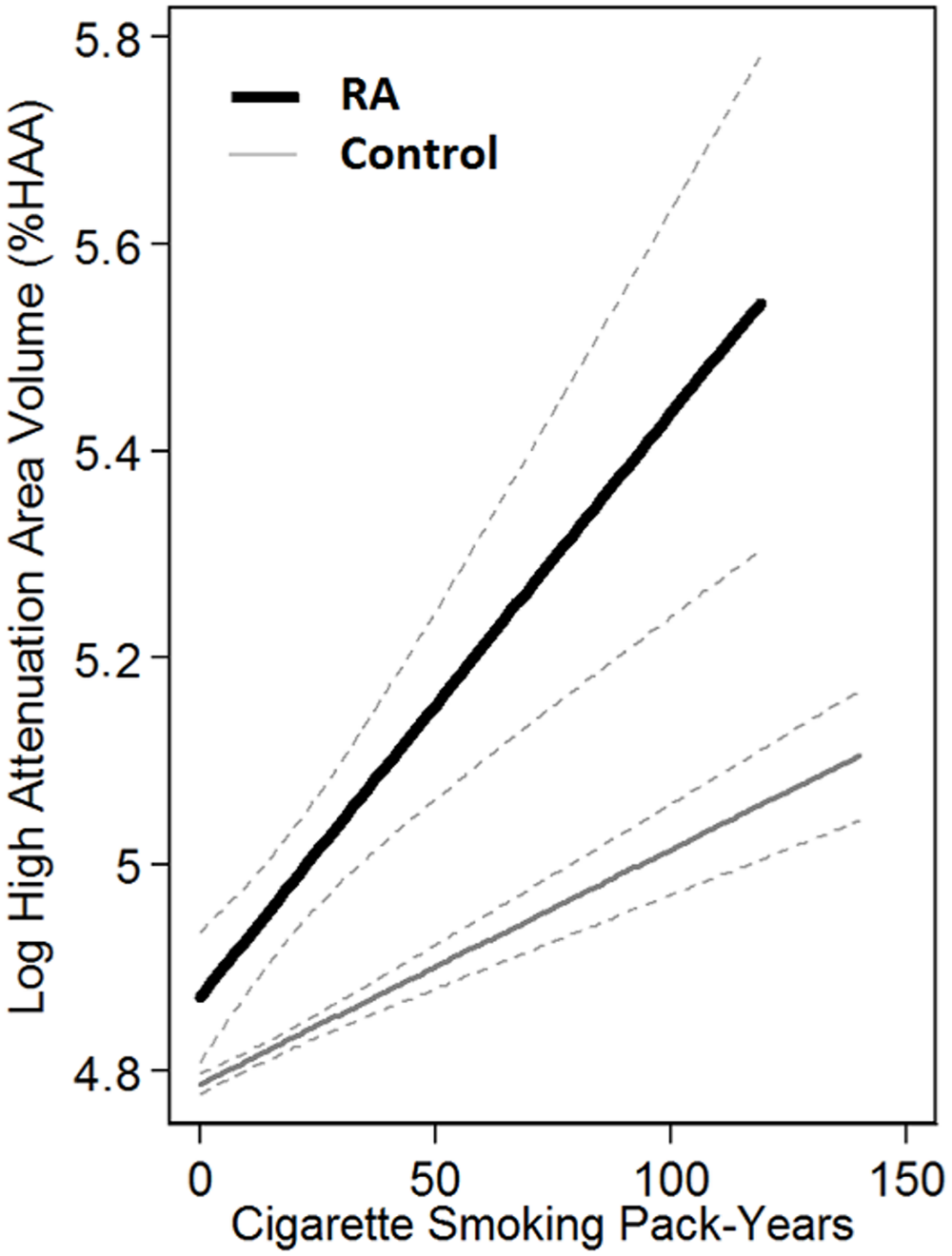
Association between Cigarette Pack-Years and Log High Attenuation Volume.

Areas of high attenuation were incrementally higher with higher cigarette smoking exposure in each group (Fig 1, S1 and S2 Figs)). After adjusting for demography, anthropometrics, and total lung volume imaged, the volume of high attenuation areas (HAA) was 0.03% (95% CI, 0.007–0.05%) higher for each 10 cigarette pack-year increase in smoking in the RA group compared with 0.008% (95% CI, 0.003–0.01%) in the non-RA group (interaction *p* = 0.001) (Table 3). After further adjustment for percent emphysema, this association was attenuated to 0.02% (95% CI, 0.001–0.04%) in the RA group and 0.005% (95% CI, 0.001–0.009%) in the non-RA group (interaction *p* = 0.01) (Table 4).

**Table 3:** CT Densitometry by Smoking History.

	Cigarette Pack-Years				Effect Estimate per 10 Pack-Yrs. (95% CI)	*P* Value
	0	1–10	11–20	>20		
ESCAPE RA						
No. of Subjects	67	23	17	58		
HAA Volume, cm^3^	134 (±43)	148 (±44)	151 (±60)	176 (±68)	0.03% (0.007–0.05)	0.01
Controls						
No. of Subjects	2,119	601	344	742		
HAA Volume, cm^3^	124 (±55)	128 (±20)	132 (±74)	139 (±61)	0.008% (0.003–0.01)	0.001

Primary Analysis. The full multivariate model includes age, sex, race/ethnicity, smoking status, height, body mass index, hip circumference, waist circumference, and total volume of imaged lung.

**Table 4:** CT Densitometry by Smoking History, Controlled for Emphysema%.

	Cigarette Pack-Years				Effect Estimate per 10 Pack-Yrs. (95% CI)	*P* Value
	0	1–10	11–20	>20		
ESCAPE RA						
No. of Subjects	67	23	17	58		
HAA Volume, cm^3^	134 (±43)	148 (±44)	151 (±60)	176 (±68)	0.02% (0.001–0.04)	0.04
Controls						
No. of Subjects	2,119	601	344	742		
HAA Volume, cm^3^	124 (±55)	128 (±20)	132 (±74)	139 (±61)	0.005% (0.001–0.009)	0.009

Secondary Analysis. The full multivariate model includes age, sex, race/ethnicity, smoking status, height, body mass index, hip circumference, waist circumference, total volume of imaged lung, and emphysema %.

Patients from the RA cohort were stratified based on the presence or absence of HLA-DRB1 shared epitope (SE) alleles. After controlling for the same covariates as the unstratified model, areas of high attenuation were higher with greater cigarette smoking exposure in the SE positive group only (Table 5). HAA was 0.03% (95% CI, 0.006–0.06%) higher for each 10 cigarette pack-year increase in smoking in the SE positive RA group compared with 0.008% (95% CI, 0.003–0.01%) in the non-RA group (interaction *p* = 0.002).

**Table 5:** Shared Epitope Allele Stratified Model.

Group	N	Effect Estimate per 10 Pack-Years (95% CI)	*P* Value
**ESCAPE RA**:			
Any SE	119	0.03% (0.006–0.06)	0.02
No SE	46	0.009% (-0.06–0.08)	0.78
**Controls**	3927	0.008% (0.003–0.01)	<0.01

The full multivariate model includes age, sex, race/ethnicity, smoking status, height, body mass index, hip circumference, waist circumference, & total volume of imaged lung.

SE: HLA-DRB1 Shared Epitope Allele.

## Discussion

In this study we compared the association of cumulative cigarette smoking with areas of high lung attenuation on CT in a large number of individuals with RA (ESCAPE RA) and generally healthy controls (MESA). We observed a higher proportion of high attenuation areas on cardiac CT with higher levels of cigarette smoke exposure. This association was significantly greater among patients with RA than in the general population. Our findings support the hypothesis that cigarette smoking acts synergistically with other host factors in RA patients to increase the risk of ILD.

Plausible biologic explanations for synergistic interactions with smoking include the antibody and genetic background of RA patients. Anticyclic citrullinated peptide antibodies (ACPA) are highly specific for RA and serum levels correlate with disease severity and response to therapy [33, 34]. Citrullination, a posttranslational protein modification, is present in the lung tissue of subjects with RA and certain citrullinated protein isoforms are highly specific for RA-ILD [33, 35, 36]. RA related lung abnormalities have been noted in patients with RA-specific autoantibodies in the serum and sputum prior to the onset of inflammatory arthritis [37, 38]. This has led to the hypothesis that the lung is the site of development or sequestering of RA autoantibodies [36, 37, 39]. Smoking enhances inflammatory cell recruitment, promoting pulmonary epithelial and endothelial injury [15, 40]. Through this process lung tissue citrullination is activated via altered expression of peptidylarginine deiminase (PAD) enzymes [1, 40, 41]. The presence of smoking induced lung citrullination may lead to abnormal to lung injury repair increasing the risk of ILD in patients with RA [10].

Patients subtyped by ACPA positivity and negativity demonstrate distinct patterns of genetic variation and disease risk profiles by smoking status [16, 19]. For example, HLA-DRB1 shared epitope alleles show strong gene-environment interactions with smoking and are found exclusively in those with anticitrulline autoimmunity [16, 19]. Furthermore, smoking is a risk factor for RA in ACPA positive, but not ACPA negative RA patients [34]. We found that the association between higher cigarette smoke exposure and greater areas of high attenuation was restricted to the group of RA patients with any SE only. This suggests that smoking, in addition to potentially causing local injury and aberrant healing, interacts with individual genetic variation to cause systemic immune system dysregulation.

Our study has multiple strengths including large, well-characterized study populations and similar protocols for data collection and MDCT acquisition. Several limitations do warrant discussion. The study has the typical limitations of observational design and most importantly cannot determine causality. Although ILD typically predominates in the lung bases, important information including concomitant emphysema could be lost from the excluded lung apices, which may be particularly relevant here as the magnitude of the association for HAA was attenuated after controlling for percent emphysema. Given the significantly higher cumulative cigarette pack-years and percent emphysema in the RA group, the presence of this data could have further attenuated the differences between the two cohorts. As previously mentioned, however, cardiac CT scans are correlated with full-lung CT scans for both measures in MESA [23, 28]. Corresponding baseline visit PFTs were not available for the ESCAPE RA group to confirm a parallel association with spirometric restriction or reduced static lung volumes. The increased volume of high lung attenuation areas with greater cigarette smoking exposure captured in our study may represent areas of lung parenchyma inflammation and/or fibrosis that correspond with interstitial lung disease[23]. Areas of high attenuation noted on MDCT, however, could represent a range of pulmonary abnormalities including small airways inflammation and thickening and pulmonary edema [23, 37] that have noted to occur in increased frequency in smokers and patients with RA. The absolute change in the volume of areas of high attenuation with greater smoking was relatively small in both cohorts. The clinical significance of this degree of change in patients without overt symptoms is unclear. Finally, the interaction between any SE and the association between cigarette smoking and HAA on CT is based on a small number of patients and warrants additional study in a larger RA cohort.

In conclusion, we found a stronger association between cigarette smoking and HAA on CT in those with RA compared with the general population. Areas of increased HAA on CT correlate with parenchymal inflammation and fibrosis and may represent subclinical ILD. Smoking is likely just one of many environmental exposures that act in combination with host (genetic) factors to initiate RA-ILD. Cigarette smoking, however, represents a major preventable risk factor for the development of RA-ILD and cessation efforts should be pursued aggressively.

## Supporting Information

S1 Data Anonymized Minimal Dataset. (CSV)

S1 Fig Scatterplot of the Association between Cigarette Pack-Years and Log High Attenuation Volume in MESA Participants. (TIF)

S2 Fig Scatterplot of the Association between Cigarette Pack-Years and Log High Attenuation Volume in ESCAPE RA Participants. (TIF)
