# Supplementary figures and images for: Smoking and Subclinical ILD in RA versus the Multi-Ethnic Study of Atherosclerosis
Source: PLoS One. 2016 Apr 6;11(4):e0153024. doi: 10.1371/journal.pone.0153024 (PMC4822776; doi:10.1371/journal.pone.0153024)

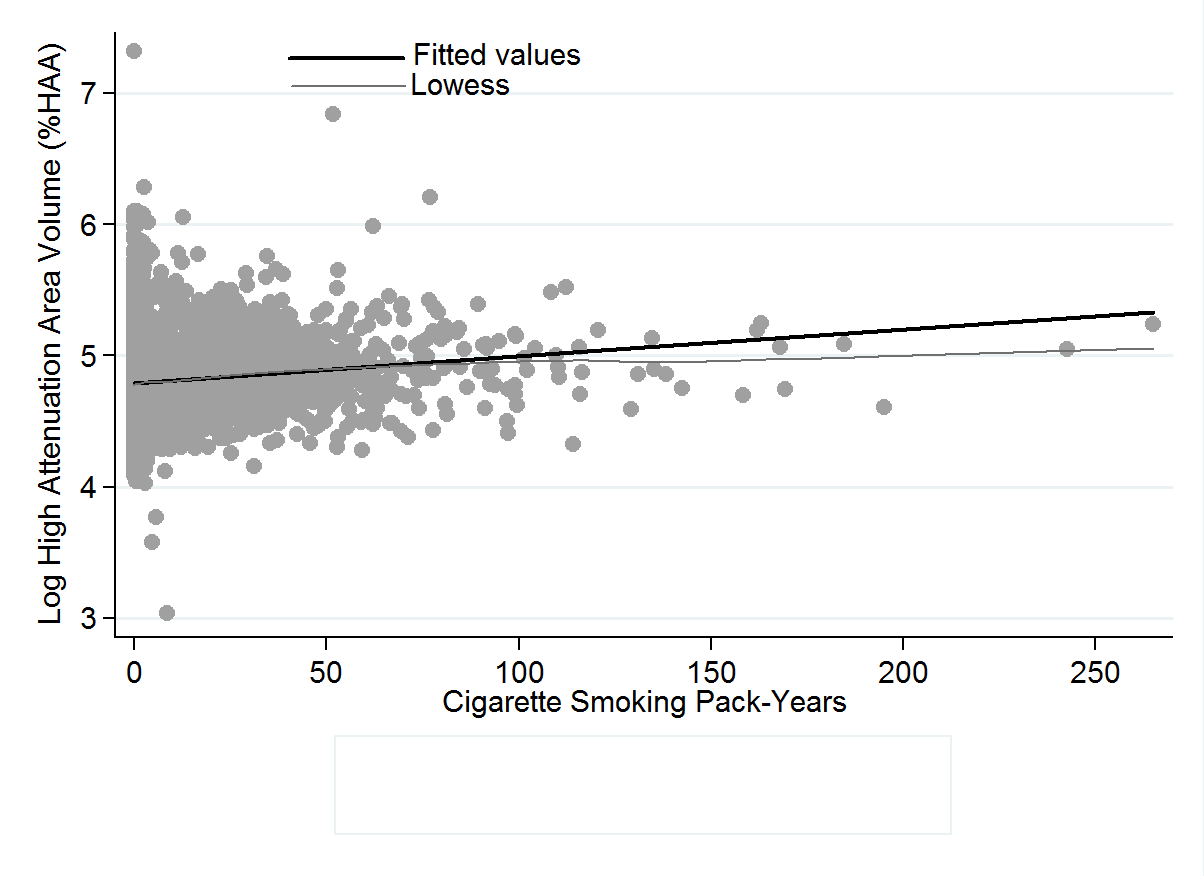

Supplement: S1 Fig — (TIF) [file pone.0153024.s002.tif]

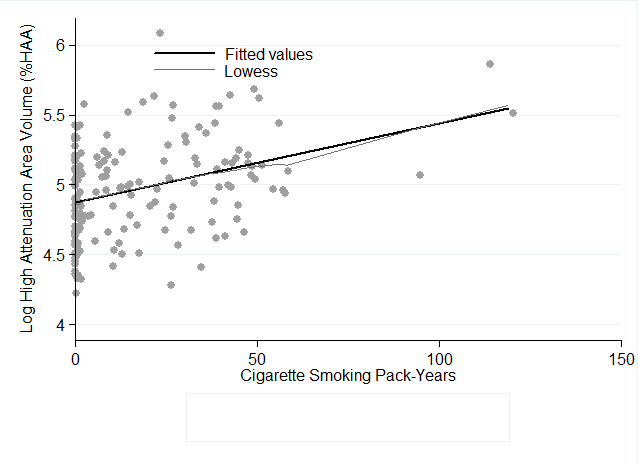

Supplement: S2 Fig — (TIF) [file pone.0153024.s003.tif]
